# Boosting the clinical use of ground reaction forces in anterior cruciate ligament injury prevention: The ‘*CUTtheACL*’ study

**DOI:** 10.1002/ksa.70017

**Published:** 2025-08-29

**Authors:** Stefano Di Paolo, Matthew Buckthorpe, Luca Pirli Capitani, Luca Ciampone, Alfredo Bravo‐Sànchez, Margherita Mendicino, Filippo Tosarelli, Alberto Grassi, Stefano Zaffagnini, Francesco Della Villa

**Affiliations:** ^1^ 2nd Orthopaedic and Traumatologic Clinic, IRCCS Istituto Ortopedico Rizzoli Bologna Italy; ^2^ Education and Research Department Isokinetic Medical Group, FIFA Medical Centre of Excellence Bologna Italy; ^3^ St Mary's University Faculty of Sport Technology and Health Sciences London UK; ^4^ Universidad Francisco de Vitoria Faculty of Health Sciences Madrid Spain; ^5^ Department of Biomedical and Neuromotor Sciences University of Bologna Bologna Italy

**Keywords:** 2D video‐analysis, ACL, cut manoeuvre, football, ground reaction forces, return to sport

## Abstract

**Purpose:**

Ground reaction forces (GRF) data have been introduced to enhance the understanding of anterior cruciate ligament (ACL) injury pathomechanics. However, translating GRF information into daily clinical practice remains challenging for high‐demanding movements such as cutting manoeuvres. This study aims to describe GRF of the 90° change of direction (COD) task, providing robust benchmark data and force‐time curve description to enhance their use in clinical practice, particularly ACL injury prevention.

**Methods:**

One thousand and two healthy football (soccer) players (16.3 ± 2.8 years, 264 females) performed three preplanned 90° COD tasks per limb at maximum intensity, with the cutting foot contact performed on an artificial turf floor embedded force platform (AMTI), which collected GRF data (frequency: 1000 Hz). Peak GRF (impact and propulsion phases, and their ratio), ground contact time, rate of force acceptance (RFA), impulse were presented as absolute values and normalised to body weight (BW). Differences in kinetics metrics according to sex, level of play, limb dominance were determined via Student's *t*‐test (*p* < 0.05). Multiple linear regression analyses determined the association between players' characteristics and kinetics.

**Results:**

Six thousand and eight valid attempts were included. Vertical GRF was 1516 ± 526 N (2.48 ± 0.79 N/BW) and occurred 32.6 ms after initial contact (10.4% of the cut stance). Vertical RFA was 95,200 ± 48,138 N/s (155.6 ± 75.5 N/s/BW). Male players had higher absolute and normalised GRFs and vertical RFA than females (*p* < 0.001). Elite players had smaller impulse: propulsion ratio than subelite players (*p* < 0.001). The regression showed limited variance of GRF metrics (adjusted‐*R* ^2^ = 0.047–0.014, *p* < 0.001).

**Conclusion:**

Normative data and explanation of clinically relevant GRF features were provided. GRF features could enrich the understanding of players' COD movement quality and performances. Sports medical and performance practitioners may include the analysis of GRF during COD as part of athlete screening for participation, injury risk and return to play, potentially offering insights for ACL (re)injury risk mitigation.

**Level of Evidence:**

Level IV, cohort study.

**Abbreviations**
ACL: anterior cruciate ligament
BMI: body mass index
BW: body weight
COD: change of direction
GCT: ground contact time
GRF: ground reaction force
IC: initial contact
KAM: knee abduction moment
RFA: rate of force acceptance
RFD: rate of force development
RTS: return to sport

## INTRODUCTION

Anterior cruciate ligament (ACL) injury rate in football (soccer) is on the rise and young players (12–18 years old) seem the most affected [15, 34, 48]. Injury risk mitigation strategies increasingly rely on the understanding of ACL injury pathomechanics and data‐driven functional testing [8, 17, 23]. However, translating the multitude of biomechanics information into daily clinical practice remains challenging, especially when it comes to high intensity, dynamic, multidirectional movements such as change of direction (COD) tasks [17, 18, 23, 26]. In this scenario, an underrated potential lies in the clinical use of ground reaction forces (GRF) during these tasks [18].

GRF metric analysis has been successfully integrated in the assessment of jump and landing performance: ground contact time (GCT), rate of force development and acceptance (RFD and RFA, derivate of force‐time curve), impulse (area under the force‐time curve) and so on, showed the potential to detect signs of poor neuromotor function and performance [28, 29, 30, 33]. Few studies have considered GRF metrics during cutting/COD manoeuvres [35, 36]. Robust benchmark data and a clear description of force‐time curves in COD tasks is thus absent. Sports medicine and performance practitioners would greatly benefit from a comprehensive understanding of GRF metrics in COD tasks to enhance their integration in ACL injury prevention and rehabilitation protocols [18, 23, 38].

The aim of the present study was to describe the GRFs and the associated kinetic metrics (GCT, RFA, impulse) of the 90° COD task. The analysis was conducted on an extensive dataset (>6000 trials) of competitive healthy football players within the ‘*CUTtheACL*’ project, a prospective epidemiology and biomechanics investigation on risk factors for primary ACL injuries [9]. The ultimate goal is to provide the sports medicine and performance practitioners with applied information to integrate force platforms in their clinical routine for the assessment of high‐demanding movement tasks and offer insights for ACL (re)injury risk mitigation.

## MATERIALS AND METHODS

### Study population

The ‘*CUTtheACL*’ study is a prospective epidemiology and biomechanics investigation on risk factors for primary ACL injury. The analysis was conducted in the Education and Research Department of Isokinetic Medical Group (Bologna, Italy). In brief, the overall project aims to prospectively assess the risk of ACL injury in young competitive football players after a baseline screening of cut manoeuvre through a qualitative 2D video‐analysis scoring system.

A total of 1002 football players belonging to elite (*n* = 286) and subelite (*n* = 716) football team academies participated in the study. Inclusion criteria were aged between 14 and 21 years with Tegner activity level ≥7. Exclusion criteria were: (1) evidence of musculoskeletal disorders or functional impairment; (2) body mass index (BMI) > 35; (3) cardiopulmonary or cardiovascular disorders and (4) inability to perform the required tasks.

For each player, the following information was collected before the COD tests: sex, age, body mass, dominant limb, first team level (elite, subelite), level of aggressiveness in the field (self‐assessed by each player, ranked through a Likert scale from 1 = minimally aggressive to 5 = extremely aggressive). The mean age was 16.3 ± 2.8 years; female players represented more than one‐fourth of the cohort (*n* = 264, Table 1).

**Table 1:** Demographics for the CUTtheACL study cohort.

	All (*n* = 1002)	Female (*n* = 264)	Male (*n* = 738)	Effect size^a^	*p* value
Age (years)	16.3 ± 2.8	18.0 ± 3.2	15.7 ± 2.3	0.89	<0.001
Body mass (kg)	62.9 ± 10.1	59.4 ± 8.6	64.1 ± 10.4	0.47	<0.001
Height (cm)	169.9 ± 9.7	162.1 ± 6.8	172.7 ± 9.0	1.25	<0.001
BMI	21.7 ± 2.8	22.6 ± 2.9	21.4 ± 2.7	0.42	<0.001
Preferred limb^b^					
Left	162 (16.2)	33 (12.5)	129 (17.5)	3.56	n. s.
Right	840 (83.8)	231 (87.5)	609 (82.5)		
Team level^c^					
Elite	286 (28.5)	74 (28.0)	212 (28.7)	0.05	n. s.
Subelite	716 (71.5)	190 (72.0)	526 (71.3)		
Aggressiveness					
1 (min)	26 (2.6)	9 (3.4)	17 (2.3)	5.84	n. s.
2	119 (11.9)	38 (14.5)	81 (11.0)		
3	440 (44.1)	102 (38.9)	338 (45.9)		
4	331 (33.2)	88 (33.6)	243 (33)		
5 (max)	82 (8.2)	25 (9.5)	57 (7.7)		

*Note*: Data are presented as mean ± standard deviation (95% confidence intervals) for continuous variables, count (percentage) for categorical variables.

^a^ Cohen's *d* (continuous variables), *Χ*² (categorical variables).

^b^ Preferred limb is intended as the kicking limb.

^c^ The first team level.

### Study procedure

**Figure 1:**
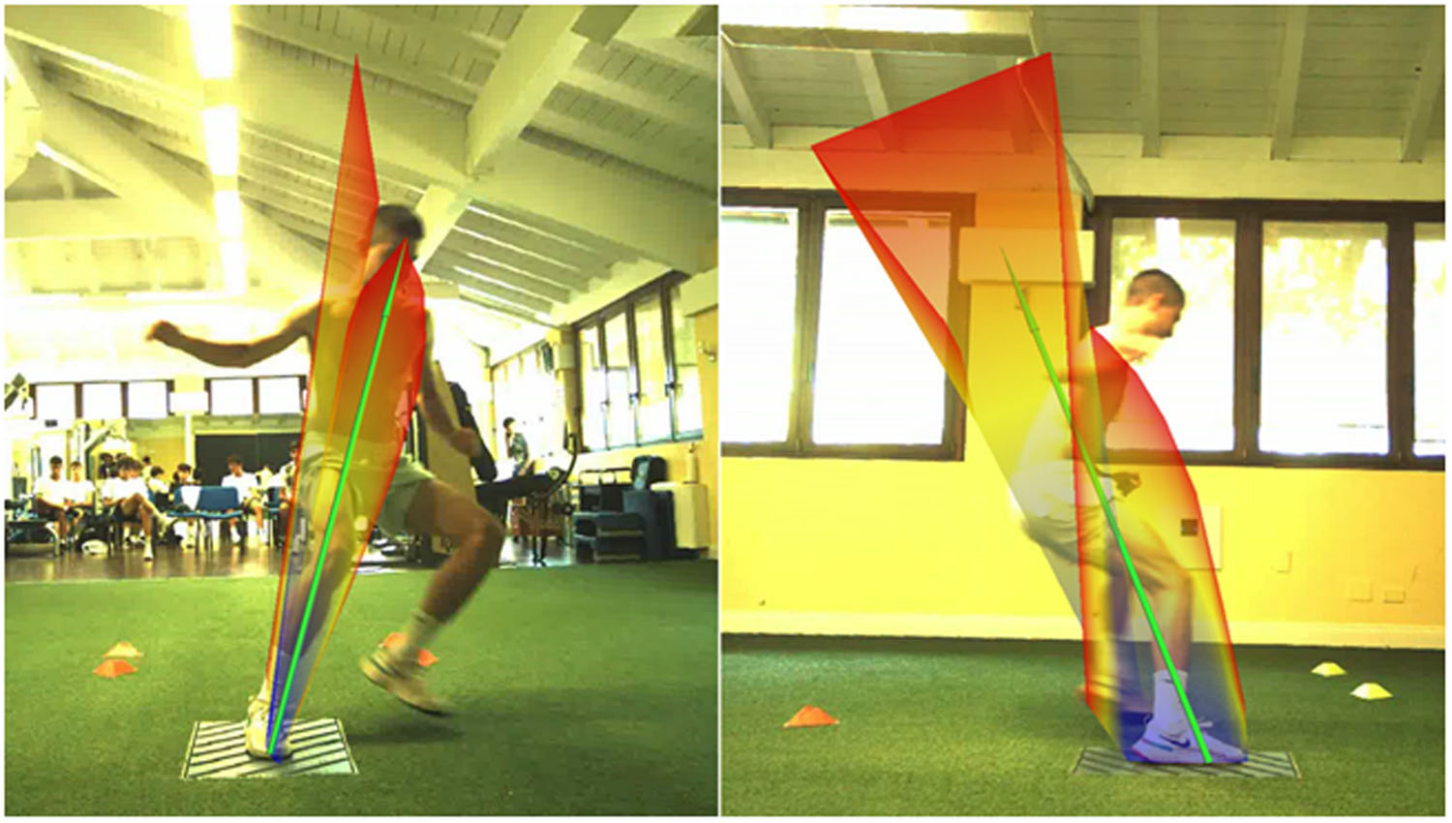
Ground reaction forces (GRF) butterfly graph for a 90° change of direction. Visual inspection of GRF in frontal (left) and sagittal (right) view could offer a first description of the change of direction movement quality and potential injury risk patterns.

Each football player was asked to perform preplanned 90° COD tasks. The laboratory floor was equipped with artificial turf, the cut direction was traced with cones (Figure 1). The complete acquisition setting has been presented in previous studies [7, 9]. In brief, each trial consisted of the player accelerating linearly forward to the force plate from a distance of 5 m, with the intention to perform a 90° COD, with reacceleration and subsequent deceleration (3 m). Players were asked to complete the movements at the maximum intensity and were given due rest if fatigued (typical time between trials: 30 s, desired rest: 1–2 min). Before the test, the participants performed a 10‐min dynamic warm‐up (exercise bike and mobility) and performed familiarisation attempts of the movement at submaximal efforts. All players performed three maximal valid left and right leg CODs. The players wore own running shoes; no cleats were allowed to minimise biases in shoe type and consistency with previous literature. Full foot contact on the force platform and satisfactory performance of a 90° cut angle were required to consider a trial valid. A floor embedded force platform (AMTI 400*600) was used to collect GRF data. Sampling frequency was 1000 Hz.

### Data Processing

**Figure 2:**
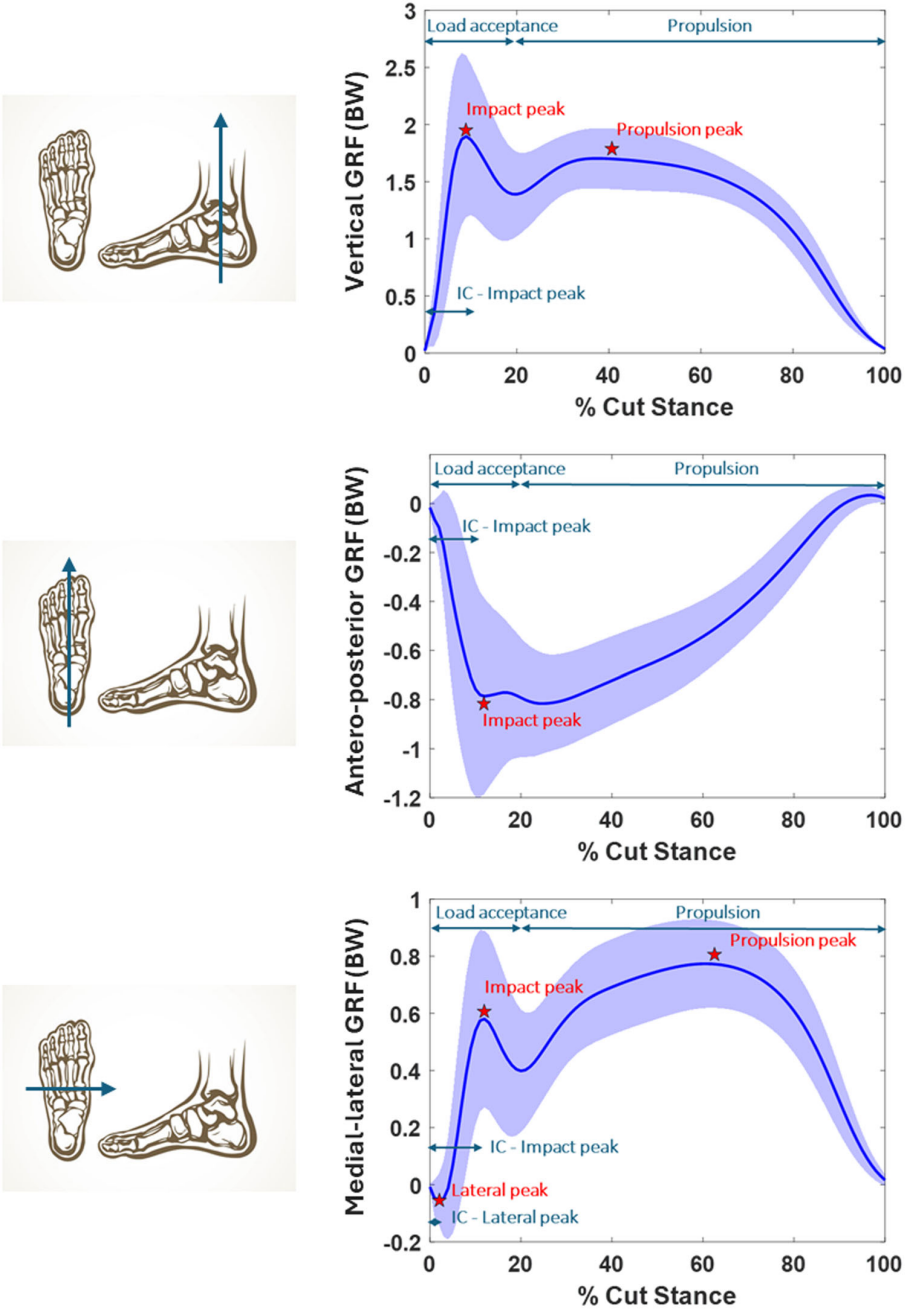
Average ground reaction forces (GRF) for a 90° change of direction in the vertical (top), anterior‐posterior (middle) and medial‐lateral (bottom) components. Solid blue line represent mean over 6008 trials and shaded area represents standard deviation. Impact and propulsion peaks are highlighted with red stars; load acceptance and propulsion areas of the curve and initial contact (IC) to impact (and lateral) peak distance are highlighted with blue arrows. GRF are normalised on players' body weight (BW).

For the purpose of this study, the force platform data were extracted from VICON Nexus (Vicon Motion Systems Ltd.) and processed in Matlab (v2023b, The MathWorks). The vertical (vGRF), anterior‐posterior (pGRF) and medial‐lateral (mGRF) components of the GRF were extracted. The vGRF component was positively defined; the pGRF was defined positive (+) for anterior propulsion force and negative (–) for braking forces; the mGRF force was defined positive (+) for forces directed towards the cut medial‐lateral direction and negative (–) for forces directed opposite to the medial‐lateral direction. The GRF curves were divided in two distinct phases according to the current literature: [14, 27] a ‘load acceptance’ phase, from the initial contact of the foot on the force platform to the local minimum in the curve (midstance), and a ‘propulsion’ phase, from the local minimum to the end of the curve. The peak GRF value in each phase was extracted and named ‘impact peak’ in the load acceptance phase and ‘propulsion peak’ in the propulsion phase. The time from initial foot contact on the force platform (IC) to the first peak, midstance and second peak were collected for each of the three GRF components. Furthermore, a third peak was noted in medial‐lateral GRF: the negative peak (i.e., contralateral to the cut direction) occurring close in time to the IC was identified as ‘lateral mGRF peak’ (Figure 2). The GCT spent on the force platform, the time from IC to the first peak, and the time and percentage of cut stance for load acceptance and propulsion phases were extracted.

The rate of force acceptance (RFA) from the IC to the first peak (moving window: 10 ms) was also computed to measure effectiveness in force absorption through eccentric muscle contraction and the impulse was computed to provide a metric of change in momentum [4, 47].

The GRF, RFA and impulse metrics were presented both absolute and normalised to players' body weight (BW). The impact: propulsion ratio was also computed for vGRF, mGRF, GCT and impulse.

### Statistical analysis

The normal distribution of the data for each variable was confirmed through the Shapiro‐Wilk test and homogeneity of variance was confirmed through Levene's test. Regarding the normative data, the continuous variables were presented as mean ± standard deviation (95% confidence interval [CI]), while the categorical variables were presented as a percentage over the total and median with interquartile range.

The two‐tailed Student's *t*‐test was used to assess normally distributed variables differences between male and female, dominant and nondominant limb, and elite and subelite players. The Cohen's d effect size and the mean difference between the groups (with 95% CI) were reported alongside the *p*‐value. The effect size was considered trivial, small, medium and large for Cohen's *d* value of <0.2, 0.2, 0.5 and 0.8, respectively. The chi‐squared test was used to inspect differences in categorical variables. The within‐subject variability was assessed through standard deviation and coefficient of variation (% standard deviation/mean) out of the six valid trials of each participant.

Multivariate linear regression analyses were performed to inspect the influence of sex, limb dominance, level of playing, family history of ACL injury (factors), age, aggressiveness (covariates) on vGRF impact peak, vRFA and impulse. Adjusted *R* ^2^ was presented alongside *p*‐values for each of the three regression models. Differences were considered statistically significant for *p* < 0.05. The statistical analyses were conducted in Matlab.

## RESULTS

Overall, 6008 valid trials were included in the final analysis. Four trials were excluded due to technical issues of the force platform. Median (interquartile range [IQR]) number of trials performed was 6 [6, 9]. Male and female players differed in age, height, body mass and BMI (Table 1).

### Ground reaction force and ground reaction time

**Figure 3:**
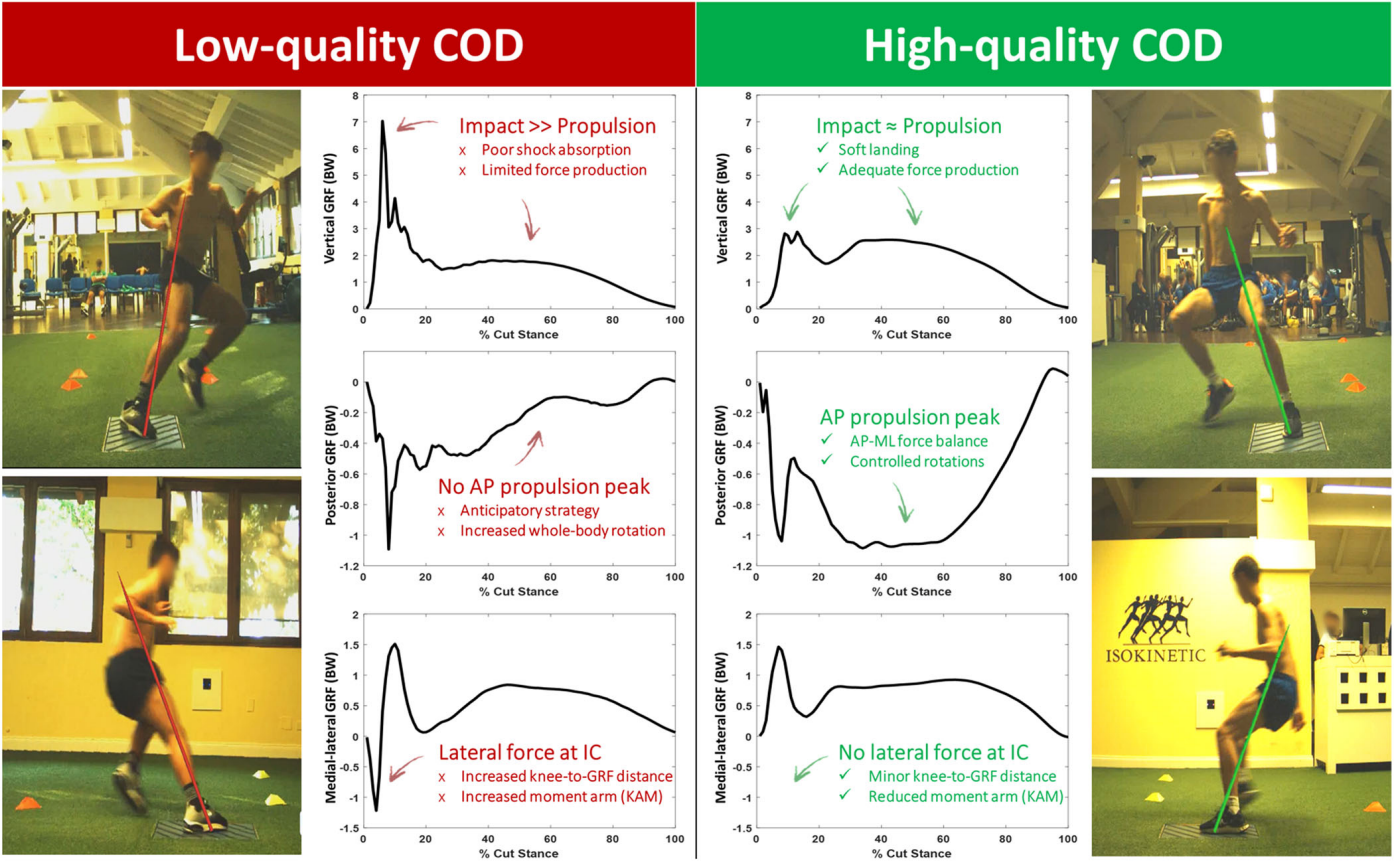
Example of ground reaction forces (GRF) curves for a 90° change of direction in the vertical (top), anterior‐posterior (middle) and medial‐lateral (bottom) components. Left‐side player is thought to put a greater load on the anterior cruciate ligament (high impact, anticipatory strategy and lateral force at initial contact) than right‐side player (soft impact, no anticipatory strategy, no lateral force at initial contact). AP, anterior‐posterior; BW, body weight; IC, initial contact; KAM, knee abduction moment; ML, medial‐lateral.

The average vGRF was 1516 ± 528 N, equating to 2.48 ± 0.79 N/BW (Table 2). The pGRF and mGRF impact peaks were less than half of the vGRF impact peak. Male players displayed higher absolute and normalised GRFs than females (vGRF diff = 0.26 N/BW, *p* < 0.001, Supporting Information S1: Appendix A1.1). Elite players displayed lower impact and higher propulsion vGRF and mGRF than subelite players (*p* < 0.001, Supporting Information S1: Appendix A1.2). No differences in GRFs between dominant and nondominant limb were found (diff = 0.2 N/BW, *p* > 0.05, Supporting Information S1: Appendix A1.3). Impact: propulsion ratio was lower for elite (1.29) than subelite players (1.42, *p* < 0.001, Supporting Information S1: Appendices A2.1 and A2.2). Examples of different GRF curve shapes with clinically relevant implications were provided for each of the three GRF components (Figure 3).

**Table 2:** Average and normalised ground reaction forces and ground reaction time for a 90° change of direction task.

Ground reaction force	Absolute (N)		Normalised (N/BW)	
	Mean ± SD	95% CI	Mean ± SD	95% CI
Impact peak vGRF	1516.4 ± 527.5	[1502.1; 1530.6]	2.48 ± 0.79	[2.46; 2.50]
Propulsion peak vGRF	1104.3 ± 232.3	[1098.1; 1110.6]	1.80 ± 0.28	[1.79; 1.81]
Impact peak pGRF	−728.8 ± 267.9	[−736.0; −721.6]	−1.19 ± 0.42	[−1.20; −1.18]
Lateral peak mGRF	−82.5 ± 87.1	[−84.8; −80.2]	−0.14 ± 0.14	[−0.14; −0.14]
Impact peak mGRF	502.7 ± 205.3	[497.2; 508.3]	0.82 ± 0.31	[0.81; 0.83]
Propulsion peak mGRF	482.5 ± 138.2	[478.8; 486.2]	0.79 ± 0.19	[0.78; 0.80]
**Ground contact time**	**Completion time (ms)**		**% Cut stance**	
	**Mean **±** SD**	**95% CI**	**Mean **±** SD**	**95% CI**
Total cut time	317.4 ± 58.2	[315.8; 319.0]		
Load acceptance phase	74.7 ± 42.1	[73.5; 75.8]	23.8 ± 12.7	[23.5; 24.2]
Propulsion phase	242.7 ± 63.6	[241.0; 244.4]	76.2 ± 12.7	[75.8; 76.5]
Impact peak vGRF	32.6 ± 15.1	[32.2; 33.0]	10.4 ± 4.7	[10.3; 10.6]
Propulsion peak vGRF	130.7 ± 49.6	[129.4; 132.1]	41.5 ± 12.9	[41.1; 41.8]
Impact peak pGRF	48.0 ± 25.8	[47.3; 48.7]	15.6 ± 9.0	[15.3; 15.8]
Lateral peak mGRF	20.3 ± 56.2	[18.8; 21.8]	6.3 ± 16.2	[5.8; 6.7]
Impact peak mGRF	37.4 ± 10.2	[37.2; 37.7]	12.1 ± 3.7	[12.0; 12.2]
Propulsion peak mGRF	151.7 ± 65.1	[150.0; 153.5]	47.9 ± 16.9	[47.4; 48.4]

Abbreviations: BW, bodyweight; CI, confidence intervals; GRF, ground reaction force; m, medial; N, Newton; p, posterior; SD, standard deviation; v, vertical.

The GCT during COD was 317.4 ± 58.2 ms. Load and acceptance phases were 23.8% and 76.2% of the cut stance, respectively (Table 2). vGRF impact peak occurred on average at 32.6 ms (10.4% of the cut stance) after initial contact. Elite players had shorter GCTs during the COD than subelite players, respectively (19 ms lower total time, *p* < 0.001, Supporting Information S1: Appendices A1.1 and A1.2).

The multivariate linear regression showed that male sex, lower age, subelite player level and higher aggressiveness were statistically predictors of higher impact vGRF, although not clinically relevant as per variance explained (adjusted‐*R* ^2^ = 0.047, *p* < 0.001).

### Rate of force acceptance and impulse

The average vRFA was 95,200 ± 48,138 N/s (155.6 ± 75.5 N/s/BW) and the load acceptance impulse was 64.4 ± 44.6 Ns (0.10 ± 0.07 Ns/BW, Table 3). Male players showed higher absolute and normalised vRFA (*d* > 0.30, *p* < 0.001) than female players (Supporting Information S1: Appendix A1.1).

**Table 3:** Average and normalised rate of force acceptance from initial contact to impact peak and impulse (work) for a 90° change of direction task.

	Absolute (N/s)		Normalised (N/s/BW)	
	Mean ± SD	95% CI	Mean ± SD	95% CI
Rate of force acceptance				
Impact peak vGRF	95,200 ± 48,138	[93,906; 96,495]	155.6 ± 75.5	[153.5; 157.6]
Impact peak pGRF	22,105 ± 17,103	[21,645; 22,565]	36.5 ± 28.5	[35.7; 37.3]
Impact peak mGRF	39,900 ± 21,873	[39,312; 40,488]	65.2 ± 34.4	[64.3; 66.2]
Impulse				
Total	251.8 ± 62.0	[250.1; 253.5]	0.41 ± 0.07	[0.41; 0.41]
Load acceptance phase	64.4 ± 44.6	[63.2; 65.6]	0.10 ± 0.07	[0.10; 0.10]
Propulsion phase	187.4 ± 65.1	[185.7; 189.2]	0.30 ± 0.09	[0.30; 0.30]

Abbreviations: BW, bodyweight; CI, confidence intervals; GRF, ground reaction force; m, medial; N, Newton; p, posterior; SD, standard deviation; v, vertical.

Propulsion and total impulse were higher in males than female players (*p* < 0.001, Appendix A1.1), while no differences were found in load acceptance impulse. Load acceptance and total impulse were higher in subelite players than elite players (*p* < 0.001, Appendix A1.2).

The multivariate linear regression showed that male sex, lower age, subelite player level and higher aggressiveness were statistically significant predictors of higher vRFA, although not clinically relevant as per variance explained (adjusted‐*R* ^2^ = 0.038, *p* < 0.001) and that family history of ACL injury and subelite player level was associated with high impulse (adjusted‐*R* ^2^ = 0.014, *p* < 0.001).

## DISCUSSION

The most important finding of the present study was the description of the GRFs of the 90° COD task from both numerical and graphical perspectives. The GRF and associated metrics (GCT, RFA, impulse) of the 90° COD task were inspected according to players' characteristics in the largest dataset so far in the literature (>1000 players, >6000 trials). The present paper aimed to provide normative data for the 90° COD kinetics that could be extracted from a force platform only. Such information may be beneficial both in football players' screening and primary prevention for ACL injury, and during the rehabilitation and return to sport (RTS) continuum to inspect the regaining of normality range of GRF, RFA and so on.

The GRF curves were analysed according to the load acceptance (shock absorption) and propulsion (force production) phases. In each phase, a peak force could be identified. The impact vGRF peak was the highest in magnitude (2.48 N/BW on average) and was in line with previous studies inspecting 90° and 110° COD tasks kinetics [12, 44]. The propulsion peak vGRF was on average 39.4% lower than the impact peak (0.7x N/BW). Uniquely, we reported and described the nonnegligible mGRF peak components. Other than the impact and propulsion peak, an initial (contra) lateral mGRF peak could be noted. The RFA and impulse were also presented as metrics of force absorption and dissipation [4]. The present analysis therefore provides both a set of metrics deducible immediately after a COD test through graphical inspection and a subset of metrics requiring data postprocessing, offering a comprehensive view of the 90° COD GRF profile.

Male players exhibited higher GRFs and longer GCTs than females during the CODs. Although a higher absolute force is not surprising, there were still higher values for males when expressed in relation to BW (although with a smaller effect size, males 11% higher). The differences in GCT were due to the longer propulsion phase for males than female players (+19 ms), while impact phase was comparable (−1 ms). Moreover, vRFA and impulse were greater for males than females (8%–19%). This could be related to a more performance‐oriented approach in young male football. It has been shown that male players have higher functional demand than female players in training and game, with females having lower capacity to sustain such rapid decelerative demands at both professional and youth levels [3, 37]. Further research should quantify differences in peak force metrics in relation to strength capacity (e.g., ratio of COD loads and peak lower limb force capabilities, such as isometric mid‐thigh pull or squat assessment). When testing players in 90° COD, a vGRF of 2.3 and 2.6 N/BW for female and male, respectively can be considered as normative values, with a sex‐related difference of 0.3x N/BW expected.

Players belonging to an elite football team exhibited lower impact forces and higher propulsion forces than subelite players both in vertical and medial‐lateral directions. Thus, elite players showed a lower impact: propulsion ratio than subelite players (1.29 vs. 1.42). The higher propulsion impulse for elite than subelite players suggests a higher movement efficiency in the former. This suggests elite players approach the COD at greater speeds, are able to eccentrically accept more load, do it with lower peaks, and produce subsequently higher propulsion forces, suggestive of enhanced stretch‐shortening cycle function and COD performance. All these resulted in a GCT 19 ms less in elite versus subelite players. GRF features related to the propulsion phase, for example, the force production peak, could be therefore considered as indicators of better performance during the 90° COD and indicate a more efficient management of the injury‐performance conflict in elite players than subelite counterparts [13, 14].

The multiple regression analysis failed to identify factors explaining variance in GRF outcomes (vGRF, vRFA, impulse, Supporting Information S1: Appendices A3.1 and A3.2). Despite being significant (*p* < 0.05), adjusted‐*R* ^2^ for the multiple regression analyses ranged between 0.014 and 0.047, thus, potentially indicating low clinical utility. According to the analysis, being a male player, younger, with higher aggressiveness, and playing in a subelite team was associated with higher impact peak vGRF, vRFA and load acceptance impulse. Although players with such characteristics are considered at risk of sustaining an ACL injury [8, 11], further studies are required to understand clinically relevant factors associated with COD kinetics, including, e.g., the occurrence of noncontact ACL injuries, players’ neuromuscular performance and braking technique.

The vGRF impact: propulsion was on average 1.39, but with variance between individuals (coefficient of variation = 33.2%, Figure 3). One in six (16%) trials had a peak impact: propulsion force <1. A high impact to propulsion peak ratio implies a difference in braking (or deceleration) and propulsion (or acceleration) features during COD. A very high vGRF (up to 7 N/BW) indicates a reduced ability of the neuromuscular system to eccentrically absorb force, leading to a more rapid and less controlled dissipation of forces and/or an increased decelerative demand on the final step, likely due to suboptimal deceleration during penultimate and antepenultimate steps [13, 14]. Research has shown higher final step forces when penultimate step forces are lower [13, 20]. In these cases, players may dissipate the body momentum during a single final step (‘tall‐thin’ impulse shape [20]). These higher peak forces during the decelerative aspect of the COD would expose the lower limb to higher and potentially dangerous forces, which would need to be accepted via the passive restraints (e.g., tendon, ligament, joint). From a kinematic perspective, a higher vGRF has been associated with reduced knee and hip flexion (stiff landing strategy) and increased foot dorsiflexion (heel strike pattern) [6, 21, 32].

The vGRF curve may also provide implications for COD performance, since a high impact peak that cannot be translated into high propulsion describes a less efficient force production and sprint in the cut direction. According to this study results (Supporting Information S1: Appenidx A1.2), high propulsion peak vGRF (>1.83 N/BW) is advisable when inspecting players' progress during (p)rehabilitation. The ratio between impact and propulsion peak vGRF could also be a surrogate metrics for players' deceleration/acceleration strategy. Further research is warranted to understand the factors associated with high and low impact: propulsion ratios and the clinical implication in COD performance analysis and injury prevention.

The pGRF is usually adopted to describe the braking forces in unidirectional movements (sprints, running). However, due to the multidirectional nature of the 90° COD, this parameter provides potentially interesting information regarding the player's strategy during the COD. Despite a potentially equal force exerted in the first peak, the propulsion phase could be either absent in AP direction (middle Figure 3, red graph) or present a peak (middle Figure 3, green graph). In the former case, no force exerted in AP direction means that the player has already turned into the new direction of movement, that is, describing an anticipatory strategy; [39] in the latter case, the player spends more time absorbing the forces in the sagittal plane before redirecting one's body mass. On the one hand, anticipatory cut strategies have been associated with higher COD performances but with an increased risk of inducing rotational components in the whole body, resulting in higher risk of ACL overload [14, 26]. Conversely, optimal shock absorption in sagittal plane is targeted during the RTS phase to enhance movement quality and reduce the risk of re‐injury to the ACL [9, 31, 42, 46]. Despite previous research has underlined the relation between high braking forces and overuse injuries in running [40], it is important to contrast horizontal kinetic features with kinematics to delineate the clinical relevance towards COD movement quality in (ACL) injury prevention.

The mGRF is often neglected in biomechanical assessments due to its lower magnitude compared to the vGRF and pGRF. However, medial‐lateral forces play a pivotal role in 90° COD movements: being either ipsilateral or contralateral to the cut direction, these forces contribute to generating external knee abduction moment (KAM) by acting on the knee moment arm (Figure 3). The KAM has been identified as the strongest biomechanical predictor of primary and secondary ACL injury occurrence [24, 25, 41]. In particular, the first contralateral (negative) peak occurs 20.3 ms after the initial contact and could be seen as a surrogate metrics for KAM. Changes in lateral GRF have been found to explain a significant portion of KAM and knee joint load distribution variance in gait [1, 5, 45]. In high‐dynamics movements, for example, decelerations and cuts, such an association has been identified by Havens and Sigward, Jones et al. and Donelon et al. [11, 16, 25, 44], for what concerns both magnitude and timing of the KAM. Despite the absence of prospective studies and the need of further research, the presence of a high lateral peak immediately after the initial contact could been argued as a potential risk factor for high KAM and subsequent risk for ACL injury. On the other hand, higher mGRF propulsion force could be linked to better capacity of sprinting in the new direction, being an indication of improved performance. In the present study, elite players showed higher mGRF than their nonelite counterparts (Supporting Information S1: Appendix A1.2).

The movement task under investigation was a preplanned COD, according to a validated protocol [7, 9]. Recent literature is suggesting the adoption of unplanned COD tasks to challenge the players also from a neurocognitive perspective and highlight potential risk factors for ACL injury [10, 19, 22, 49]. Future work should include the assessment of planned and unplanned COD tasks with the addition of sport‐specific elements to mimic in‐game situations and improve the understanding of clinically relevant variations in GRF features [2, 19].

The present study has some limitations. First, the biomechanical analysis was conducted with a cross‐sectional design, so no day‐to‐day differences from relevant football season time points (during competitive season vs. season break) could be inspected [43]. Within‐participant variability was presented to provide a further clinically useful dispersion metric based on individual performance (Supporting Information S1: Appendix A4). Second, no EMG data were collected through the tests to minimise the setup complexity. Such data could have offered interesting insights into the joint and muscle force absorption and production in response to the GRFs, paving the way for musculoskeletal modelling simulations. Last two limitations come from the cohort under investigation. The players were all relatively young (mean age 16.3 years) at the time of the test. Therefore, normative data most reliably apply to young population and might not be generalisable to adult (professional) football players. Furthermore, the players were all uninjured at the time of the test. Thus, no difference between injured and noninjured players is possible at the current state of the project and optimal rehabilitation progression cannot be determined through such an analysis. Future works on the *CUTtheACL* study will focus on the prospective assessment of players biomechanics according to the current injury surveillance.

The clinical relevance of the present study is the production of robust benchmark data for 90° COD task GRF and associated kinetic variables that could be used by sports medicine and performance practitioners involved in football players' testing. Moreover, the description of the force‐time curves presented could help practitioners in the interpretation of the COD movement quality immediately after a test, with no need for technical skills and long postprocessing time.

Differences in GRF metrics within and between the players might be adopted to inspect the eccentric and concentric capacities of the players towards both ACL injury prevention, RTS continuum and performance analysis. Such practical information could help boost the clinical use of GRFs in daily clinical environments in the testing of football players. Further studies based on such a benchmark might allow the understanding of the risk factors for primary and secondary ACL injury prevention [38].

## CONCLUSION

Normative data and explanation of clinically relevant GRF features were provided. Vertical and medio‐lateral GRFs and RFA could highlight poor neuromuscular control, movement strategies that put high load on the ACL and discriminate between male/female and elite/subelite COD performances. Sports medicine and performance practitioners may include the analysis of GRF in the clinical routine for the assessment of high‐demanding movement tasks and offer insights for ACL injury prevention.

## AUTHOR CONTRIBUTIONS

Stefano Di Paolo and Francesco Della Villa conceived the study. Luca P. Capitani, Luca Ciampone, Filippo Tosarelli and Alfredo Bravo‐Sànchez conducted the data collection. Stefano Di Paolo and Margherita Mendicino performed the data analysis, tables/figures designs and statistical analysis. Stefano Di Paolo, Matthew Buckthorpe and Alberto Grassi contributed to data interpretation. Stefano Di Paolo and Matthew Buckthorpe drafted the manuscript. Francesco Della Villa and Stefano Zaffagnini supervised the study and provided the equipment. All the authors revised and approved the final version.

## CONFLICT OF INTEREST STATEMENT

Each author certifies that he or she has no commercial associations (e.g., consultancies, stock ownership, equity interest, patent/licensing arrangements, etc) that might pose a conflict of interest in connection with the submitted article.

## ETHICS STATEMENT

This study obtained the approval from Institutional Review Board (IRB approval: n. 283861 of 04/11/2021) of Bioethical Committee of University of Bologna. All the participants or their legal tutor signed informed consent before entering the study.

## Supporting information

Appendix.

## Data Availability

The data that support the findings of this study are available from the corresponding author upon reasonable request.
