# Supplementary material for: Boosting the clinical use of ground reaction forces in anterior cruciate ligament injury prevention: The ‘CUTtheACL’ study
Source: Knee Surg Sports Traumatol Arthrosc. 2025 Aug 29;33(11):4059–69. doi: 10.1002/ksa.70017 (PMC12582228; doi:10.1002/ksa.70017)
Supplement: Supplementary file 1 — Appendix. [file KSA-33-4059-s001.docx]

**Appendix**

**Appendix A1.1.** Differences in ground reaction force, ground reaction time, rate of force acceptance, and impulse between female and male football players. Note: GRF=Ground reaction force; N=Newton; BW=bodyweight; SD=standard deviation; CI=confidence intervals; v=vertical; p=posterior; m=medial.

| **Ground Reaction Force** | ***Absolute (N)*** | | | | |  |  | ***Normalized (N/BW)*** | | | | |
| --- | --- | --- | --- | --- | --- | --- | --- | --- | --- | --- | --- | --- |
|  | **Female** | **Male** | **Diff [95% CI]** | **Effect size** | **p-value** |  |  | **Female** | **Male** | **Diff [95% CI]** | **Effect size** | **p-value** |
| Impact peak vGRF | 1343.8 ± 467.8 | 1589.0 ± 534.3 | 245.2 [214.8; 275.5] | 0.49 | <0.001 |  |  | 2.30 ± 0.68 | 2.55 ± 0.82 | 0.26 [0.21; 0.30] | 0.34 | <0.001 |
| Propulsion peak vGRF | 1019.3 ± 183.3 | 1140.1 ± 241.4 | 120.8 [107.5; 134.1] | 0.56 | <0.001 |  |  | 1.75 ± 0.24 | 1.82 ± 0.28 | 0.07 [0.05; 0.08] | 0.26 | <0.001 |
| Impact peak pGRF | -627.4 ± 221.0 | -771.5 ± 274.4 | -144.2 [-159.5; -128.9] | 0.58 | <0.001 |  |  | -1.08 ± 0.35 | -1.24 ± 0.44 | -0.17 [-0.19; -0.14] | 0.42 | <0.001 |
| Lateral peak mGRF | -69.8 ± 68.9 | -87.9 ± 93.2 | -18.1 [-23.2; -13.0] | 0.22 | <0.001 |  |  | -0.12 ± 0.12 | -0.14 ± 0.15 | -0.02 [-0.03; -0.01] | 0.14 | <0.001 |
| Impact peak mGRF | 479.4 ± 195.6 | 512.6 ± 208.5 | 33.2 [21.1; 45.2] | 0.16 | <0.001 |  |  | 0.82 ± 0.31 | 0.82 ± 0.32 | 0.00 [-0.02; 0.02] | 0.00 | 0.899 |
| Propulsion peak mGRF | 464.6 ± 116.3 | 490.0 ± 145.8 | 25.4 [17.3; 33.5] | 0.19 | <0.001 |  |  | 0.80 ± 0.18 | 0.78 ± 0.20 | -0.02 [-0.03; -0.01] | 0.09 | 0.002 |
| **Ground Reaction Time** | ***Completion time (ms)*** | | | | |  |  | ***% Cut Stance*** | | | | |
|  | **Female** | **Male** | **Diff [95% CI]** | **Effect size** | **p-value** |  |  | **Female** | **Male** | **Diff [95% CI]** | **Effect size** | **p-value** |
| Total cut time | 304.8 ± 55.4 | 322.7 ± 58.5 | 18.0 [14.6; 21.4] | 0.32 | <0.001 |  |  |  |  |  |  |  |
| Load acceptance phase | 75.5 ± 42.2 | 74.3 ± 42.1 | -1.2 [-3.7; 1.3] | 0.03 | 0.332 |  |  | 25.2 ± 13.9 | 23.2 ± 12.1 | -2.0 [-2.7; -1.3] | 0.15 | <0.001 |
| Propulsion phase | 229.2 ± 64.0 | 248.4 ± 62.6 | 19.2 [15.5; 22.9] | 0.30 | <0.001 |  |  | 74.8 ± 13.9 | 76.8 ± 12.1 | 2.0 [1.3; 2.7] | 0.15 | <0.001 |
| Impact peak vGRF | 33.1 ± 13.1 | 32.4 ± 15.8 | -0.8 [-1.6; 0.1] | 0.05 | 0.093 |  |  | 11.1 ± 4.8 | 10.1 ± 4.6 | -1.0 [-1.3; -0.7] | 0.21 | <0.001 |
| Propulsion peak vGRF | 130.8 ± 42.3 | 130.7 ± 52.4 | -0.1 [-3.0; 2.8] | 0.00 | 0.938 |  |  | 43.3 ± 11.8 | 40.7 ± 13.2 | -2.6 [-3.4; -1.9] | 0.21 | <0.001 |
| Impact peak pGRF | 50.7 ± 26.8 | 46.9 ± 25.3 | -3.8 [-5.3; -2.3] | 0.15 | <0.001 |  |  | 17.2 ± 9.8 | 14.9 ± 8.5 | -2.3 [-2.8; -1.7] | 0.25 | <0.001 |
| Lateral peak mGRF | 30.0 ± 78.2 | 16.2 ± 43.2 | -13.8 [-17.1; -10.5] | 0.22 | <0.001 |  |  | 9.4 ± 23.4 | 4.9 ± 11.7 | -4.5 [-5.5; -3.6] | 0.25 | <0.001 |
| Impact peak mGRF | 37.0 ± 10.7 | 37.6 ± 9.9 | 0.6 [0.0; 1.2] | 0.06 | 0.039 |  |  | 12.5 ± 4.1 | 11.9 ± 3.5 | -0.5 [-0.8; -0.3] | 0.14 | <0.001 |
| Propulsion peak mGRF | 157.2 ± 61.4 | 149.4 ± 66.5 | -7.8 [-11.6; -4.0] | 0.12 | <0.001 |  |  | 51.4 ± 15.4 | 46.4 ± 17.4 | -4.9 [-5.9; -3.9] | 0.30 | <0.001 |
| **Rate of Force Acceptance** | ***Absolute (N/s)*** | | | | |  |  | ***Normalized (N/s/BW)*** | | | | |
|  | **Female** | **Male** | **Diff [95% CI]** | **Effect size** | **p-value** |  |  | **Female** | **Male** | **Diff [95% CI]** | **Effect size** | **p-value** |
| Impact peak vGRF | 80013.1 ± 39997.1 | 101591.3 ± 49817.5 | 21578.2 [18801.8; 24354.7] | 0.48 | <0.001 |  |  | 137.1 ± 64.7 | 163.3 ± 78.3 | 26.3 [21.9; 30.7] | 0.37 | <0.001 |
| Impact peak pGRF | 17300.7 ± 13575.3 | 24126.8 ± 18008.8 | 6826.2 [5835.3; 7817.1] | 0.43 | <0.001 |  |  | 30.1 ± 24.0 | 39.2 ± 29.8 | 9.2 [7.5; 10.8] | 0.34 | <0.001 |
| Impact peak mGRF | 36458.9 ± 19605.8 | 41347.8 ± 22605.4 | 4888.8 [3606.8; 6170.9] | 0.23 | <0.001 |  |  | 62.4 ± 31.9 | 66.4 ± 35.4 | 4.0 [2.0; 6.0] | 0.12 | <0.001 |
| **Impulse** | ***Absolute (Ns)*** | | | | |  |  | ***Normalized (Ns/BW)*** | | | | |
|  | **Female** | **Male** | **Diff [95% CI]** | **Effect size** | **p-value** |  |  | **Female** | **Male** | **Diff [95% CI]** | **Effect size** | **p-value** |
| Total | 226.6 ± 48.6 | 262.4 ± 63.9 | 35.7 [32.2; 39.3] | 0.63 | <0.001 |  |  | 0.39 ± 0.06 | 0.42 ± 0.07 | 0.03 [0.02; 0.03] | 0.41 | <0.001 |
| Load acceptance phase | 62.4 ± 44.7 | 65.2 ± 44.5 | 2.8 [0.2; 5.4] | 0.06 | 0.035 |  |  | 0.11 ± 0.07 | 0.10 ± 0.07 | -0.00 [-0.01; 0.00] | 0.03 | 0.324 |
| Propulsion phase | 164.3 ± 55.5 | 197.2 ± 66.3 | 32.9 [29.2; 36.7] | 0.54 | <0.001 |  |  | 0.28 ± 0.09 | 0.31 ± 0.09 | 0.03 [0.03; 0.04] | 0.34 | <0.001 |

**Appendix A1.2.** Differences in ground reaction force, ground reaction time, rate of force acceptance, and impulse between elite and sub-elite football players. Note: GRF=Ground reaction force; N=Newton; BW=bodyweight; SD=standard deviation; CI=confidence intervals; v=vertical; p=posterior; m=medial.

| **Ground Reaction Force** | ***Absolute (N)*** | | | | |  |  | ***Normalized (N/BW)*** | | | | |
| --- | --- | --- | --- | --- | --- | --- | --- | --- | --- | --- | --- | --- |
|  | **Elite** | **Sub-elite** | **Diff [95% CI]** | **Effect size** | **p-value** |  |  | **Elite** | **Sub-elite** | **Diff [95% CI]** | **Effect size** | **p-value** |
| Impact peak vGRF | 1511.5 ± 492.0 | 1517.7 ± 537.1 | 6.2 [-28.0; 40.5] | 0.01 | 0.722 |  |  | 2.33 ± 0.67 | 2.52 ± 0.81 | 0.19 [0.14; 0.24] | 0.25 | <0.001 |
| Propulsion peak vGRF | 1183.5 ± 217.8 | 1082.0 ± 231.5 | -101.5 [-116.4; -86.7] | 0.45 | <0.001 |  |  | 1.83 ± 0.26 | 1.79 ± 0.27 | -0.04 [-0.06; -0.02] | 0.16 | <0.001 |
| Impact peak pGRF | -741.0 ± 251.3 | -725.4 ± 272.4 | 15.6 [-1.8; 33.0] | 0.06 | 0.079 |  |  | -1.15 ± 0.36 | -1.21 ± 0.44 | -0.06 [-0.09; -0.03] | 0.16 | <0.001 |
| Lateral peak mGRF | -88.2 ± 92.9 | -80.9 ± 85.3 | 7.3 [1.6; 12.9] | 0.08 | 0.012 |  |  | -0.14 ± 0.14 | -0.14 ± 0.14 | 0.00 [-0.01; 0.01] | 0.01 | 0.661 |
| Impact peak mGRF | 544.4 ± 198.8 | 491.0 ± 205.6 | -53.4 [-66.6; -40.1] | 0.26 | <0.001 |  |  | 0.84 ± 0.30 | 0.81 ± 0.32 | -0.03 [-0.05; -0.01] | 0.10 | 0.003 |
| Propulsion peak mGRF | 526.0 ± 142.6 | 470.2 ± 134.5 | -55.8 [-64.6; -46.9] | 0.40 | <0.001 |  |  | 0.82 ± 0.21 | 0.78 ± 0.18 | -0.04 [-0.05; -0.03] | 0.20 | <0.001 |
| **Ground Reaction Time** | ***Completion time (ms)*** | | | | |  |  | ***% Cut Stance*** | | | | |
|  | **Elite** | **Sub-elite** | **Diff [95% CI]** | **Effect size** | **p-value** |  |  | **Elite** | **Sub-elite** | **Diff [95% CI]** | **Effect size** | **p-value** |
| Total cut time | 302.7 ± 58.2 | 321.5 ± 57.5 | 18.8 [15.1; 22.6] | 0.33 | <0.001 |  |  |  |  |  |  |  |
| Load acceptance phase | 67.9 ± 34.8 | 76.6 ± 43.8 | 8.7 [6.0; 11.4] | 0.22 | <0.001 |  |  | 22.9 ± 11.3 | 24.1 ± 13.1 | 1.2 [0.4; 2.1] | 0.10 | 0.003 |
| Propulsion phase | 234.8 ± 61.5 | 245.0 ± 64.0 | 10.1 [6.0; 14.3] | 0.16 | <0.001 |  |  | 77.1 ± 11.3 | 75.9 ± 13.1 | -1.2 [-2.1; -0.4] | 0.10 | 0.003 |
| Impact peak vGRF | 31.7 ± 15.0 | 32.8 ± 15.1 | 1.1 [0.1; 2.1] | 0.07 | 0.030 |  |  | 10.8 ± 5.3 | 10.3 ± 4.5 | -0.4 [-0.7; -0.1] | 0.09 | 0.005 |
| Propulsion peak vGRF | 122.6 ± 45.5 | 133.0 ± 50.5 | 10.4 [7.2; 13.6] | 0.22 | <0.001 |  |  | 40.9 ± 12.6 | 41.6 ± 12.9 | 0.7 [-0.1; 1.5] | 0.06 | 0.099 |
| Impact peak pGRF | 49.4 ± 26.4 | 47.6 ± 25.6 | -1.8 [-3.5; -0.1] | 0.07 | 0.038 |  |  | 16.9 ± 9.8 | 15.2 ± 8.7 | -1.7 [-2.3; -1.2] | 0.19 | <0.001 |
| Lateral peak mGRF | 28.1 ± 74.1 | 18.1 ± 49.9 | -9.9 [-13.6; -6.3] | 0.16 | <0.001 |  |  | 8.6 ± 21.3 | 5.6 ± 14.4 | -3.0 [-4.1; -2.0] | 0.17 | <0.001 |
| Impact peak mGRF | 36.2 ± 9.6 | 37.8 ± 10.3 | 1.6 [1.0; 2.3] | 0.16 | <0.001 |  |  | 12.3 ± 3.9 | 12.0 ± 3.6 | -0.3 [-0.5; -0.1] | 0.08 | 0.012 |
| Propulsion peak mGRF | 136.9 ± 59.1 | 155.9 ± 66.1 | 19.0 [14.8; 23.2] | 0.30 | <0.001 |  |  | 45.9 ± 17.6 | 48.5 ± 16.7 | 2.6 [1.5; 3.7] | 0.15 | <0.001 |
| **Rate of Force Acceptance** | ***Absolute (N/s)*** | | | | |  |  | ***Normalized (N/s/BW)*** | | | | |
|  | **Elite** | **Sub-elite** | **Diff [95% CI]** | **Effect size** | **p-value** |  |  | **Elite** | **Sub-elite** | **Diff [95% CI]** | **Effect size** | **p-value** |
| Impact peak vGRF | 95948.5 ± 46276.6 | 94989.4 ± 48653.4 | -959.1 [-4085.7; 2167.5] | 0.02 | 0.548 |  |  | 147.6 ± 66.5 | 157.8 ± 77.7 | 10.2 [5.3; 15.1] | 0.14 | <0.001 |
| Impact peak pGRF | 21600.7 ± 17494.8 | 22247.3 ± 16990.5 | 646.6 [-464.2; 1757.4] | 0.04 | 0.254 |  |  | 33.2 ± 26.1 | 37.4 ± 29.1 | 4.2 [2.4; 6.1] | 0.15 | <0.001 |
| Impact peak mGRF | 42231.9 ± 21759.0 | 39242.3 ± 21862.1 | -2989.5 [-4407.9; -1571.1] | 0.14 | <0.001 |  |  | 65.5 ± 32.6 | 65.2 ± 34.9 | -0.3 [-2.5; 1.9] | 0.01 | 0.787 |
| **Impulse** | ***Absolute (Ns)*** | | | | |  |  | ***Normalized (Ns/BW)*** | | | | |
|  | **Elite** | **Sub-elite** | **Diff [95% CI]** | **Effect size** | **p-value** |  |  | **Elite** | **Sub-elite** | **Diff [95% CI]** | **Effect size** | **p-value** |
| Total | 254.7 ± 63.5 | 251.0 ± 61.6 | -3.7 [-7.7; 0.4] | 0.06 | 0.074 |  |  | 0.39 ± 0.08 | 0.41 ± 0.07 | 0.02 [0.02; 0.03] | 0.29 | <0.001 |
| Load acceptance phase | 59.4 ± 38.9 | 65.8 ± 45.9 | 6.4 [3.5; 9.3] | 0.15 | <0.001 |  |  | 0.09 ± 0.06 | 0.11 ± 0.07 | 0.02 [0.01; 0.02] | 0.25 | <0.001 |
| Propulsion phase | 195.3 ± 64.6 | 185.2 ± 65.0 | -10.0 [-14.2; -5.8] | 0.15 | <0.001 |  |  | 0.30 ± 0.09 | 0.31 ± 0.09 | 0.00 [-0.00; 0.01] | 0.05 | 0.132 |

**Appendix A1.3.** Differences in ground reaction force, ground reaction time, rate of force acceptance, and impulse between dominant and non-dominant limb. Note: GRF=Ground reaction force; N=Newton; BW=bodyweight; SD=standard deviation; CI=confidence intervals; v=vertical; p=posterior; m=medial.

| **Ground Reaction Force** | ***Absolute (N)*** | | | | |  |  | ***Normalized (N/BW)*** | | | | |
| --- | --- | --- | --- | --- | --- | --- | --- | --- | --- | --- | --- | --- |
|  | **Dominant** | **Non-dominant** | **Diff [95% CI]** | **Effect size** | **p-value** |  |  | **Dominant** | **Non-dominant** | **Diff [95% CI]** | **Effect size** | **p-value** |
| Impact peak vGRF | 1511.8 ± 523.0 | 1520.9 ± 532.0 | 9.1 [-19.3; 37.5] | 0.02 | 0.530 |  |  | 2.47 ± 0.78 | 2.48 ± 0.80 | 0.02 [-0.02; 0.06] | 0.02 | 0.450 |
| Propulsion peak vGRF | 1101.9 ± 233.5 | 1106.7 ± 231.2 | 4.8 [-7.7; 17.3] | 0.02 | 0.454 |  |  | 1.80 ± 0.27 | 1.8 ± 0.26 | 0.01 [-0.01; 0.02] | 0.03 | 0.250 |
| Impact peak pGRF | -732.3 ± 266.1 | -725.4 ± 269.8 | 6.9 [-7.6; 21.3] | 0.03 | 0.351 |  |  | -1.20 ± 0.42 | -1.19 ± 0.43 | 0.01 [-0.01; 0.03] | 0.03 | 0.335 |
| Lateral peak mGRF | -78.8 ± 83.9 | -86.2 ± 90.1 | -7.5 [-12.1; -2.8] | 0.09 | 0.002 |  |  | -0.13 ± 0.14 | -0.14 ± 0.15 | -0.01 [-0.02; -0.00] | 0.09 | 0.002 |
| Impact peak mGRF | 501.6 ± 205.0 | 503.9 ± 205.6 | 2.3 [-8.8; 13.3] | 0.01 | 0.684 |  |  | 0.82 ± 0.31 | 0.82 ± 0.31 | 0.00 [-0.01; 0.02] | 0.01 | 0.691 |
| Propulsion peak mGRF | 478.9 ± 137.7 | 486.1 ± 138.7 | 7.2 [-0.2; 14.7] | 0.05 | 0.056 |  |  | 0.78 ± 0.19 | 0.79 ± 0.19 | 0.01 [0.00; 0.02] | 0.07 | 0.017 |
|  |  |  |  |  |  |  |  |  |  |  |  |  |
| **Ground Reaction Time** | ***Completion time (ms)*** | | | | |  |  | ***% Cut Stance*** | | | | |
|  | **Dominant** | **Non-dominant** | **Diff [95% CI]** | **Effect size** | **p-value** |  |  | **Dominant** | **Non-dominant** | **Diff [95% CI]** | **Effect size** | **p-value** |
| Total cut time | 320.9 ± 60.4 | 313.9 ± 55.7 | -6.9 [-10.0; -3.8] | 0.12 | <0.001 |  |  |  |  |  |  |  |
| Load acceptance phase | 76.0 ± 43.1 | 73.4 ± 41.2 | -2.6 [-4.9; -0.3] | 0.06 | 0.024 |  |  | 24.0 ± 12.7 | 23.7 ± 12.8 | -0.2 [-0.9; 0.4] | 0.02 | 0.481 |
| Propulsion phase | 244.9 ± 64.8 | 240.6 ± 62.3 | -4.3 [-7.7; -0.9] | 0.07 | 0.013 |  |  | 76.1 ± 12.7 | 76.3 ± 12.8 | 0.2 [-0.4; 0.9] | 0.02 | 0.481 |
| Impact peak vGRF | 33.2 ± 15.3 | 31.9 ± 14.8 | -1.3 [-2.1; -0.5] | 0.08 | 0.002 |  |  | 10.5 ± 4.7 | 10.4 ± 4.7 | -0.2 [-0.4; 0.1] | 0.04 | 0.174 |
| Propulsion peak vGRF | 131.8 ± 50.9 | 129.7 ± 48.3 | -2.1 [-4.8; 0.6] | 0.04 | 0.122 |  |  | 41.4 ± 12.9 | 41.6 ± 12.8 | 0.2 [-0.5; 0.9] | 0.02 | 0.570 |
| Impact peak pGRF | 48.4 ± 25.3 | 47.6 ± 26.3 | -0.9 [-2.2; 0.5] | 0.03 | 0.227 |  |  | 15.5 ± 8.7 | 15.6 ± 9.2 | 0.1 [-0.4; 0.5] | 0.01 | 0.811 |
| Lateral peak mGRF | 23.3 ± 63.7 | 17.3 ± 47.4 | -6.0 [-9.0; -2.9] | 0.11 | <0.001 |  |  | 7.1 ± 18.6 | 5.4 ± 13.3 | -1.8 [-2.7; -0.9] | 0.11 | <0.001 |
| Impact peak mGRF | 38.1 ± 10.4 | 36.8 ± 9.8 | -1.3 [-1.9; -0.8] | 0.13 | <0.001 |  |  | 12.2 ± 3.8 | 12.0 ± 3.6 | -0.2 [-0.4; 0.0] | 0.05 | 0.063 |
| Propulsion peak mGRF | 149.2 ± 67.1 | 154.3 ± 63.0 | 5.1 [1.6; 8.6] | 0.08 | 0.004 |  |  | 46.6 ± 17.3 | 49.2 ± 16.5 | 2.5 [1.6; 3.4] | 0.15 | <0.001 |
|  |  |  |  |  |  |  |  |  |  |  |  |  |
| **Rate of Force Acceptance** | ***Absolute (N/s)*** | | | | |  |  | ***Normalized (N/s/BW)*** | | | | |
|  | **Dominant** | **Non-dominant** | **Diff [95% CI]** | **Effect size** | **p-value** |  |  | **Dominant** | **Non-dominant** | **Diff [95% CI]** | **Effect size** | **p-value** |
| Impact peak vGRF | 93645.3 ± 47883.5 | 96754.7 ± 48350.4 | 3109.4 [520.6; 5698.1] | 0.06 | 0.019 |  |  | 152.9 ± 74.6 | 158.3 ± 76.2 | 5.4 [1.3; 9.4] | 0.07 | 0.010 |
| Impact peak pGRF | 21856.5 ± 17063.6 | 22353.5 ± 17142.1 | 497.0 [-423.1; 1417.2] | 0.03 | 0.290 |  |  | 36.1 ± 28.3 | 37.0 ± 28.7 | 0.9 [-0.6; 2.4] | 0.03 | 0.248 |
| Impact peak mGRF | 39157.2 ± 21475.1 | 40642.1 ± 22241.9 | 1484.9 [308.7; 2661.1] | 0.07 | 0.013 |  |  | 64.0 ± 33.8 | 66.5 ± 35.0 | 2.5 [0.6; 4.3] | 0.07 | 0.009 |
|  |  |  |  |  |  |  |  |  |  |  |  |  |
| **Impulse** | ***Absolute (Ns)*** | | | | |  |  | ***Normalized (Ns/BW)*** | | | | |
|  | **Dominant** | **Non-dominant** | **Diff [95% CI]** | **Effect size** | **p-value** |  |  | **Dominant** | **Non-dominant** | **Diff [95% CI]** | **Effect size** | **p-value** |
| Total | 254.6 ± 63.5 | 249.1 ± 60.4 | -5.5 [-8.8; -2.2] | 0.09 | 0.001 |  |  | 0.41 ± 0.07 | 0.40 ± 0.07 | -0.01 [-0.01; -0.00] | 0.12 | <0.001 |
| Load acceptance phase | 65.5 ± 44.8 | 63.3 ± 44.4 | -2.2 [-4.6; 0.2] | 0.05 | 0.074 |  |  | 0.11 ± 0.07 | 0.10 ± 0.07 | -0.00 [-0.01; 0.00] | 0.05 | 0.057 |
| Propulsion phase | 189.1 ± 66.2 | 185.8 ± 63.9 | -3.3 [-6.8; 0.2] | 0.05 | 0.062 |  |  | 0.31 ± 0.09 | 0.3 ± 0.09 | -0.00 [-0.01; -0.00] | 0.05 | 0.045 |

**Appendix A2.1.** Impact/Propulsion ratio. Absolute, normalized, and % differences were presented alongside impact/propulsion ratio as mean and 95% confidence intervals. Note: GRF=Ground reaction force; N=Newton; BW=bodyweight; CGT=ground contact time; CI=confidence intervals; v=vertical; m=medial.

|  | **Absolute** | **Normalized (BW)** | **% Difference** | **Impact: Propulsion Ratio** |
| --- | --- | --- | --- | --- |
| **vGRF (N, BW)** | 412.0 [399.2; 424.8] | 0.68 [0.66; 0.71] | 39.4 [38.2; 40.6] | 1.39 [1.38; 1.40] |
| **mGRF (N, BW)** | 20.3 [15.5; 25.0] | 0.03 [0.02; 0.04] | 14.5 [-1.3; 30.4] | 1.15 [0.99; 1.31] |
| **GCT (ms, %)** | -168.1 [-170.5; -165.6] | -52.34 [-53.02; -51.66] | -62.9 [-63.8; -61.9] | 0.37 [0.36; 0.38] |
| **Impulse (Ns, Ns/BW)** | -123.1 [-125.5; -120.6] | -0.20 [-0.20; -0.20] | -48.8 [-50.8; -46.7] | 0.51 [0.49; 0.53] |


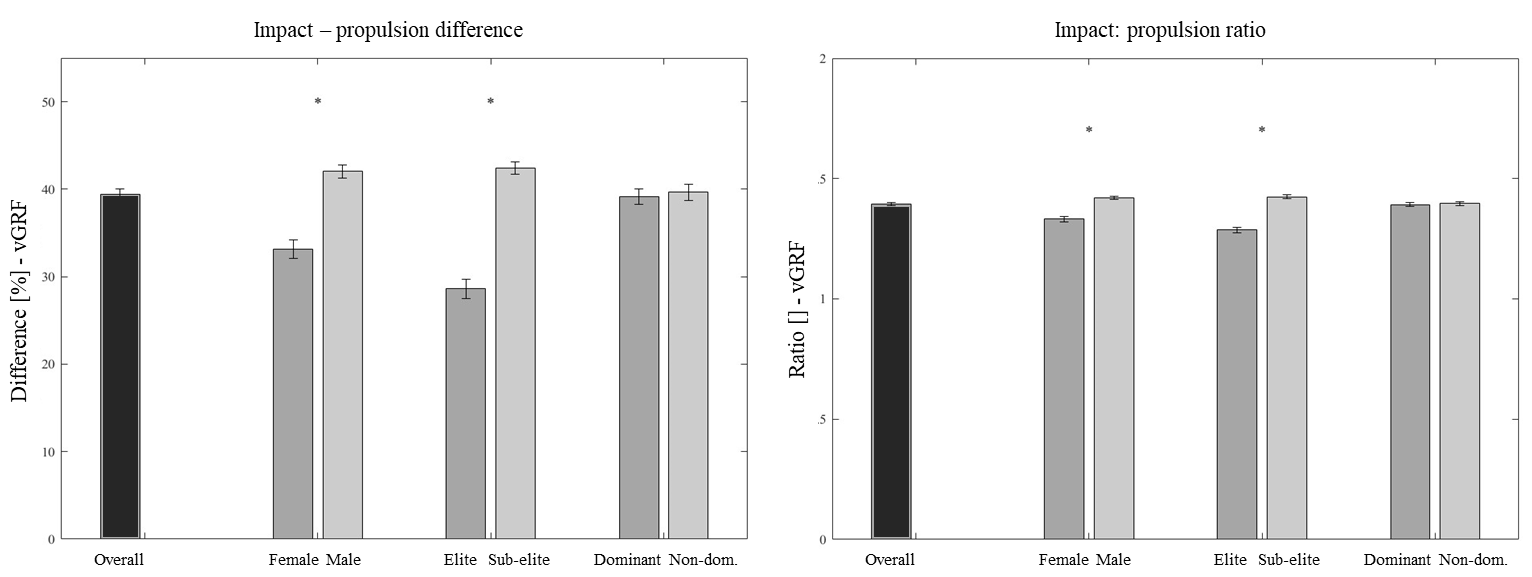


**Appendix A2.2.** Impact/Propulsion differences (left) and ratio (right) for vertical Ground reaction force. Data were presented as mean and 95% confidence intervals. Asterisks represent statistically significant differences between the groups (p<0.05).

|  | **vGRF impact peak**  adjusted-R^2^=0.047, p<0.001 | | | **vRFA**  adjusted-R^2^=0.038, p<0.001 | | | **Impulse**  adjusted-R^2^=0.014, p<0.001 | | |
| --- | --- | --- | --- | --- | --- | --- | --- | --- | --- |
| **Covariate** | **β** | **SE** | **p-value** | **β** | **SE** | **p-value** | **β** | **SE** | **p-value** |
| **Age** | -0.037 | 0.004 | < .001 | -2.843 | 0.387 | < .001 | 0.0004 | 0.0004 | 0.250 |
| **Aggressiveness** | 0.038 | 0.012 | 0.001 | 2.835 | 1.135 | 0.013 | -0.001 | 0.001 | 0.243 |
| **Gender (Male)** | 0.175 | 0.025 | < .001 | 19.584 | 2.400 | < .001 | 0.002 | 0.002 | 0.450 |
| **Team Level (Elite)** | -0.189 | 0.026 | < .001 | -9.125 | 2.491 | < .001 | -0.020 | 0.002 | < .001 |
| **Dominant limb (R)** | 0.018 | 0.021 | 0.400 | 5.458 | 2.034 | 0.007 | -0.003 | 0.002 | 0.119 |
| **ACL inj. History (Y)** | 0.012 | 0.028 | 0.671 | 0.841 | 2.641 | 0.750 | 0.005 | 0.003 | 0.032 |

**Appendix A3.1.** Multivariate linear regression models for three relevant GRF metrics. Note: GRF=Ground reaction force; RFA=rate of force acceptance; v=vertical; SE=standard error

***vGRF****= 2.86 - 0.037*****Age*** *+ 0.038*****Aggressiveness*** *+ 0.175*****Sex(M)*** *- 0.189*****Team Level(E)*** *+ 0.018*****Dominant limb(R)*** *+ 0.012*****ACL inj. History***

***vRFA****=178.16 - 2.843*****Age*** *+ 2.835*****Aggressiveness*** *+ 19.584*****Sex(M)*** *- 9.125*****Team Level(E)*** *+ 5.458*****Dominant limb(R)*** *+ 0.841*****ACL inj. history***

***Impulse****=0.189 - 0.0004*****Age*** *- 0.0001*****Aggressiveness*** *+ 0.002*****Sex(M)*** *- 0.020*****Team Level(E)*** *- 0.003*****Dominant limb(R)*** *+ 0.005*****ACL inj. history***

**Appendix A3.2.** Multivariate linear regression equations

| **Ground Reaction Force** | ***Absolute (N)*** | |  |  | ***Normalized (N/BW)*** | |
| --- | --- | --- | --- | --- | --- | --- |
|  | **CV (%)** | **SD (N)** |  |  | **CV (%)** | **SD (N/BW)** |
| Impact peak vGRF | 17.4 [12.9; 23.3] | 255.4 [185.6; 344.4] |  |  | 17.4 [12.9; 23.3] | 0.4 [0.3; 0.6]] |
| Propulsion peak vGRF | 8.2 [5.8; 11.3] | 87.1 [62.2; 124.8] |  |  | 8.2 [5.8; 11.3] | 0.2 [0.1; 0.2] |
| Impact peak pGRF | 18.6 [24.5; 86.7] | 129.5 [83.6; 189.2] |  |  | 18.6 [24.5; 13.4] | 0.2 [0.1; 0.3]] |
| Lateral peak mGRF | 62.6 [22.5; 13.9] | 43.1 [23.4; 70.9] |  |  | 62.6 [86.7; 43.0] | 0.1 [0.0; 0.1] |
| Impact peak mGRF | 22.5 [16.3; 29.9] | 107.8 [80.2; 144.7] |  |  | 22.5 [16.3; 29.9] | 0.2 [0.1; 0.2] |
| Propulsion peak mGRF | 13.9 [9.8; 20.0] | 65.4 [46.4; 96.2] |  |  | 13.9 [9.8; 20.0] | 0.1 [0.1; 0.2] |
|  |  |  |  |  |  |  |
| **Ground Reaction Time** | ***Completion time (ms)*** | |  |  | ***% Cut Stance*** | |
|  | **CV (%)** | **SD (ms)** |  |  | **CV (%)** | **SD** |
| Total cut time | 9.23 [6.8; 11.7] | 28.7 [20.7; 37.9] |  |  |  |  |
| Load acceptance phase | 29.2 [11.6; 54.7] | 16.5 [6.9; 47.8] |  |  | 29.1 [14.0; 55.0] | 5.7 [2.7; 15.9] |
| Propulsion phase | 15.0 [10.3; 24.6] | 37.7 [25.2; 56.0] |  |  | 7.1 [3.4; 22.1] | 5.7 [2.7; 15.9] |
| Impact peak vGRF | 23.8 [16.2; 36.4] | 7.0 [4.6; 11.4] |  |  | 24.2 [16.9; 36.9] | 2.4 [1.5; 3.9] |
| Propulsion peak vGRF | 23.6 [13.7; 34.9] | 29.8 [16.3; 47.7] |  |  | 23.1 [14.9; 32.9] | 10.0 [6.0; 13.8] |
| Impact peak pGRF | 33.9 [20.3; 48.4] | 17.4 [7.4; 24.9] |  |  | 34.6 [21.2; 49.8] | 5.7 [2.5; 8.4] |
| Lateral peak mGRF | 54.8 [37.3; 76.3] | 5.2 [3.4; 7.9] |  |  | 54.2 [37.9; 77.7] | 1.7 [1.1; 2.5] |
| Impact peak mGRF | 16.9 [12.2; 24.3] | 6.4 [4.4; 9.2] |  |  | 18.4 [13.4; 25.0] | 2.2 [1.5; 3.0] |
| Propulsion peak mGRF | 29.5 [19.5; 41.3] | 43.6 [27.2; 63.2] |  |  | 27.9 [15.7; 39.7] | 13.2 [7.5; 18.0] |
|  |  |  |  |  |  |  |
| **Rate of Force Acceptance** | ***Absolute (N/s)*** | |  |  | ***Normalized (N/s/BW)*** | |
|  | **CV (%)** | **SD (N/s)** |  |  | **CV (%)** | **SD (N/s/BW)** |
| Impact peak vGRF | 27.7 [20.4; 36.2] | 24071.6 [16177.0; 35636.3] |  |  | 27.7 [20.4; 36.2] | 40.2 [26.7; 56.0] |
| Impact peak pGRF | 50.7 [39.0; 64.4] | 9764.7 [6286.5; 14198.2] |  |  | 50.7 [39.0; 64.4] | 15.9 [10.3; 23.2] |
| Impact peak mGRF | 33.0 [24.0; 43.0] | 11923.8 [8304.5; 16646.7] |  |  | 33.0 [24.0; 43.0] | 19.7 [14.3; 26.8] |
|  |  |  |  |  |  |  |
| **Impulse** | ***Absolute (Ns)*** | |  |  | ***Normalized (Ns/BW)*** | |
|  | **CV (%)** | **SD (Ns)** |  |  | **CV (%)** | **SD (Ns/BW)** |
| Total | 8.4 [6.0; 1.4] | 20.2 [14.7; 28.1] |  |  | 8.4 [6.0; 1.4] | 0.03 [0.02; 0.05] |
| Load acceptance phase | 35.3 [15.8; 63.4] | 17.4 [8.4; 47.4] |  |  | 35.3 [15.8; 63.4] | 0.03 [0.01; 0.08] |
| Propulsion phase | 16.1 [10.2; 31.4] | 30.4 [19.2; 50.8] |  |  | 16.1 [10.2; 31.4] | 0.05 [0.03; 0.09] |
|  |  |  |  |  |  |  |
| **Impact:Propulsion Ratio** |  | |  |  |  | |
|  | **CV (%)** | **SD** |  |  |  |  |
| vGRF (N, BW) | 18.14 [13.56; 24.01] | 0.24 [0.18; 0.34] |  |  |  |  |
| mGRF (N, BW) | 23.11 [16.83; 31.86] | 0.24 [0.17; 0.33] |  |  |  |  |
| GCT (ms, %) | 35.26 [17.35; 85.56] | 0.09 [0.04; 0.49] |  |  |  |  |
| Impulse (Ns, Ns/BW) | 45.29 [22.40; 107.91] | 0.13 [0.06; 0.91] |  |  |  |  |

**Appendix A4.** Analysis of within-subject variability through coefficient of variation (CV) and standard deviation (SD) across the six valid trials of each player. Note: GRF=Ground reaction force; N=Newton; BW=bodyweight; v=vertical; p=posterior; m=medial.
